# Dorsal Luno-capitate Impingement in a Professional Tennis Player: A Case Report

**DOI:** 10.5704/MOJ.1711.002

**Published:** 2017-11

**Authors:** A Afshar, A Tabrizi

**Affiliations:** Department of Orthopaedics, Urmia University of Medical Sciences, Urmia, Iran

**Keywords:** chronic wrist pain, dorsal wrist impingement syndrome, impingement in athletes, dorsal luno-capitate impingement syndrome, lunate exostosis

## Abstract

A 30-year old male right handed professional tennis player complained about reduced athletic performance, chronic pain and restricted extension of his right wrist. Lateral radiograph of the right wrist demonstrated an osteophyte projecting from the dorsal lip of the lunate bone. The presence of an osteophyte on the lateral radiograph of the lunate along with the history, clinical examination, intra-operative findings, and post-operative satisfactory result made the diagnosis of dorsal luno-capitate impingement syndrome reasonable.

## Introduction

Evaluation of chronic wrist pain in professional athletes as well as in normal population is a challenging problem. Bone impingement syndromes, including dorsal wrist impingement syndrome, luno-triquetral impingement syndrome and the ulnar-sided wrist impaction-impingement syndromes are well-known clinical entities in the wrist region and frequently cause chronic wrist pain among the professional athletes^1-3^. The current report presents a case of dorsal impingement of the lunate-capitate in a professional tennis player. As far as is known, dorsal luno-capitate impingement syndrome has not been described previously.

## Case Report

**Fig. 1:**
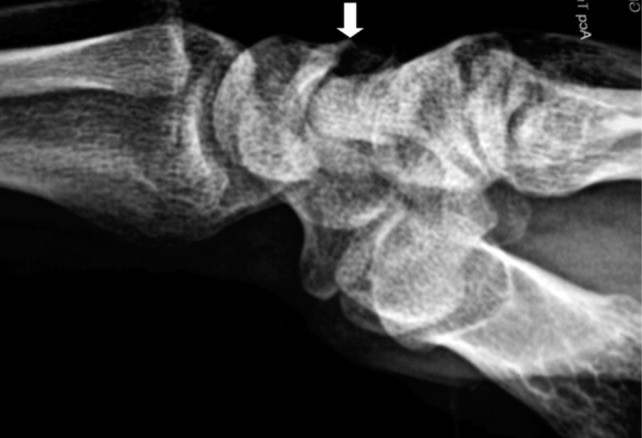
The arrow on the lateral radiograph of the right wrist indicates an osteophyte projected from the dorsal lip of the lunate bone.

A 30-year old male right handed general practitioner who was a professional tennis player complained about his reduced athletic performance, chronic pain, and restricted extension of his right wrist. The patient had been having right wrist pain for two years. The pain was progressive and intensified by provocative manoeuvre of wrist hyperextension. The right wrist extension was restricted to 20 degrees. There was tenderness on palpation of the lunate bone. The right hand power grip was 30kg while the left hands was 55kg. The lateral radiograph of right wrist demonstrated an osteophyte projecting from the dorsal lip of the lunate bone (Fig. 1).

**Fig. 2:**
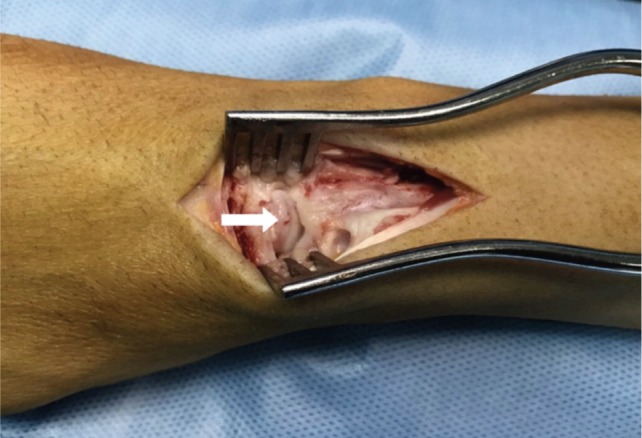
Clinical photograph showing osteophyte in the dorsal lip of the lunate, which was then debrided.

At surgery, the luno-capitate joint was evaluated by a direct dorsal approach, through which capsular thickening, synovitis, and an osteophyte projecting from the dorsal lip of the lunate bone were found. There were cartilage erosions and subchondral bone exposure on the dorsal pole of lunate bone and also on the corresponding site of capitate head. The osteophyte was removed and synovitis was debrided (Fig. 2). Histopathological study of the removed osteophyte demonstrated a normal bone structure. The wrist pain was reduced after the surgery. Three months after operation wrist extension was enhanced to 60 degrees and the right hand power grip improved to 40kg. The patient was satisfied with restoration of his right hand function.

## Discussion

The athletes’ wrists are exposed to high impact loadings, repetitive motions, axial compressions, torsional forces and distractions. History of previous injuries, improper equipment handlings and incorrect techniques predispose the athletes’ wrists to injuries and chronic wrist pain^1-3^. In the current case, the projected osteophyte on the dorsal pole of the lunate might had developed because of the chronic and repetitive micro-traumatic chondral and subchondral erosions of the proximal pole of the lunate on capitate during forceful wrist hyperextension. Chronic synovitis and capsular thickening had occurred following the impaired repair process due to repeated stress on the ligaments and joint capsule. Chronic wrist pain is a multifactorial problem^4^. Impingement syndrome due to intracapsular or extracapsular lesions can result in chronic wrist pain^4^. Dorsal wrist capsular impingement (DWCI) has been defined by Matson *et al* as impingement of dorsal capsular tissue during wrist extension, which is accompanied by chronic wrist pain^4^.

Magnetic resonance imaging is the most useful technique for detecting synovitis, capsular thickening, and chondral and subchondral erosions; however, presence of an osteophyte on the plain radiograph also makes the diagnosis of bone impingement easy^5^. Treatment of dorsal luno-capitate impingement syndrome as well as other impingement syndromes varies according to its cause. Splint, rest, nonsteroidal anti-inflammatory drugs, and corticosteroid injection may have potential benefits. The refractory cases may need synovectomy, capsulectomy and debridement of the offending osteophytes^1-3^.

The dorsal luno-capitate impingement syndrome is a distinct clinical entity different from the dorsal wrist impingement syndrome which occurs at the dorsal rim of radius and carpal bones, luno-triquetral impingement syndrome and the ulnar-sided wrist impaction-impingement syndromes (hamatolunate impingement, ulnar impaction, ulnar impingement and ulnar styloid impingement syndromes)^1-3^.

In the current case the presence of an osteophyte on the dorsal pole of the lunate was demonstrated on the lateral wrist radiograph, which along with the history, clinical examination, intra-operative findings and satisfactory postoperative result made the clinical diagnosis of dorsal lunocapitate impingement syndrome conclusive and definite.
